# Parenteral Succinate Reduces Systemic ROS Production in Septic Rats, but It Does Not Reduce Creatinine Levels

**DOI:** 10.1155/2018/1928945

**Published:** 2018-11-06

**Authors:** Sebastián P. Chapela, Isabel Burgos, Christian Congost, Romina Canzonieri, Alexis Muryan, Manuel Alonso, Carlos A. Stella

**Affiliations:** ^1^Servicio de Terapia Intensiva, Hospital Británico de Buenos Aires, Buenos Aires ZIP C1280AEB, Argentina; ^2^Departamento de Bioquímica Humana. CONICET, INBIOMED, Universidad de Buenos Aires, Facultad de Medicina, Buenos Aires ZIP C1121ABE, Argentina; ^3^Servicio de Laboratorio General, Hospital Británico de Buenos Aires, Buenos Aires ZIP C1280AEB, Argentina; ^4^Departamento de Ciencias Biológicas, Universidad de Buenos Aires, Ciclo Básico Común, Buenos Aires ZIP C1435CAE, Argentina

## Abstract

In sepsis, reactive oxygen species (ROS) production is increased. This process takes place mainly within the electron transport chain. ROS production is part of the pathophysiology of multiple organ failure in sepsis. Succinate yields Dihydroflavine-Adenine Dinucleotide (FADH_2_), which enters the chain through complex II, avoiding complex I, through which electrons are lost. The aim of this work is to determine if parenteral succinate reduces systemic ROS production and improves kidney function. Rats with cecal ligation and puncture were used as model of sepsis, and 4 groups were made: Control group; Succinate group, which only received parenteral succinate; Sepsis group; and Sepsis which received parenteral succinate. Systemic ROS are measured 24 hours after the procedure. Rats subjected to cecal puncture treated with succinate had less systemic ROS than Septic untreated rats (*p* = 0.007), while there were no differences in creatinine levels (*p* = 0.07). There was no correlation between creatinine and systemic ROS levels (*p* = 0.3). We concluded that parenteral succinate reduces ROS levels, but it does not reduce creatinine levels. Since there is no correlation between both levels, the processes would not be related.

## 1. Introduction

Sepsis is a pathology whose incidence is increasing, with a mortality rate that, according to a recent report, can reach 40% [1]. Its mortality rate is related to multiple organ failure [1].

Triggering mechanisms of organ failure appear to be multiple. Oxidative stress seems to be one of these triggering mechanisms. There are different definitions of oxidative stress, but the most common and descriptive one is the imbalance between the production of reactive oxygen species (ROS) and cellular antioxidant capacity, which can potentially damage cells and destroy tissue [2].

Reactive oxygen species are a group of molecules which include oxygen radicals, such as superoxide (O_2_^·−^), hydroxyl (OH^·^), peroxide (RO_2_^·^), and alkoxide (RO^·^), as well as nonradicals that are oxidizing agents or that quickly become radicals, such as hypochlorous acid (HOCl), singlet oxygen (^1^O_2_), and hydrogen peroxide (H_2_O_2_) [3]. On the other hand, there are also reactive nitrogen species (RNS), both radical and nonradical, such as nitric oxide (NO), peroxynitrite (ONOO^−^), and nitrogen dioxide (NO_2_^·^) [4, 5].

Over the last few years, there have been a large number of studies that describe oxidative stress in patients with sepsis, with evidence of ROS production, related damage, and antioxidant depletion [6]. Serious conditions are characterized by hyperinflammation, cellular immune dysfunction, oxidative stress, and oxidative mitochondrial dysfunction [7]. An activated immune system and mitochondrial dysfunction are the two most powerful sources of reactive molecular species. It has been proven that sepsis is characterized by an increase in the production of ROS, as well as reactive nitrogen species (RNS), both in circulation (produced by cells of the immune system and the endothelial system) and tissue (due to mitochondrial dysfunction and the modification of the antioxidant state) [4, 5].

When antioxidant defenses are outnumbered, oxidative stress, which can significantly damage lipids, proteins, and nucleic acids, occurs both within the mitochondria and the rest of the cell [2, 6, 7].

During sepsis, most ROS are produced in the mitochondria. It is believed that this is due to the electron loss that takes place when they are passed from complex I to complex III of the mitochondrial electron transport chain. On the other hand, the uncoupling of the chain also takes place between complex I and complex II [8]. Electrons that reduce complex I come from reduced coenzymes, Nicotinamide adenine dinucleotide (NADH+H^+^), while Dihydroflavine-Adenine Dinucleotide (FADH), which comes from succinate oxidation in the Krebs cycle, is oxidized in complex II, with electrons entering through the said complex [8]. In different studies, succinate has improved oxygen consumption in septic rat muscle [8], prolonging the survival of the said septic animals [9] and improving the hepatic metabolic profile [10].

The objective of this study is to observe whether the administration of intraperitoneal succinate to rats subjected to cecal ligation and puncture reduces ROS production and improves sepsis-induced kidney failure.

## 2. Materials and Methods

### 2.1. Animals

Male Sprague Dawley rats of 200 grams of average weight adapted to 12 h light cycles for 7 days and fed *ad libitum* at a room temperature of 24°C were used. The experiments were approved by the IACUC (Institutional Animal Care and Use Committee) of the Faculty of Medicine of the University of Buenos Aires, Argentina.

### 2.2. Groups

**Figure 1:**
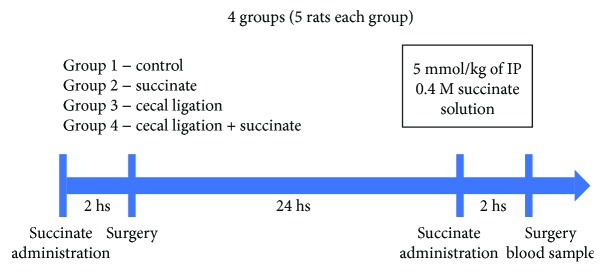
Procedure flow chart. Surgery was made only in groups 3 and 4. Succinate was administered in groups 2 and 4.

Four groups were formed: (1) Control group; (2) Succinate group, to which intraperitoneal succinate was administered 2 hours before initiating the surgery of groups 3 and 4 and 2 hours before the taking of the sample; (3) Cecal Puncture group, on which the procedure described in the following item was performed; (4) Cecal Puncture and Succinate group, to which succinate was administered 2 hours before the surgery and 2 hours before the taking of the sample. 24 hours passed between the surgery and the taking of the sample (Figure 1).

### 2.3. Cecal Ligation and Puncture

The procedure was performed under sedation and anesthesia with 100 mg/kg of intraperitoneal Ketamine and 2.5 mg/kg of intraperitoneal Xylazine. In accordance with the technique described in literature [11–16], a midline laparotomy was performed, the cecum was identified, and 1 cm was ligated. Both sides of the ligated cecum were punctured with a 25 × 8 needle, and subsequently, a layered ligation of abdominal wall was performed. A single dose of 30 mg/kg of intraperitoneal ceftriaxone and 25 mg/kg of clindamycin was administrated and then 20 ml/kg of intraperitoneal NaCl 0.9%.

### 2.4. Succinate Solution

Intraperitoneal succinate solution was administrated on groups 2 and 4, according to the flow chart specifications. 5 mmol/kg of intraperitoneal succinate 0.4 M solution was administered. Solution was prepared from succinic acid (Sigma Chemical Co.), neutralized with NaOH, and sterilized by filtration.

### 2.5. Procedure

Intraperitoneal succinate solution was administered to rats of groups 2 and 4. Two hours later, cecal ligation and puncture was performed to rats of groups 3 and 4. 24 hours later, another administration of intraperitoneal succinate solution was performed to rats of groups 2 and 4. Two hours later, blood samples were taken, and rats were sacrificed after that (Figure 1).

### 2.6. Blood Sample

A blood sample was taken through cardiac puncture under anesthesia with 100 mg/kg of intraperitoneal Ketamine and 2.5 mg/kg of intraperitoneal Xylazine, and the animal was later euthanized. Blood was centrifuged in a dry tube at 3000 rpm (900g) for 5 minutes. Then, the serum was separated and frozen at −75°C until measurement.

### 2.7. Measurement of Systemic ROS

The measurement of systemic ROS was taken using 2′,7′ dichlorofluorescein-diacetate (DCFH) (Sigma Chemical Co.). 12 *μ*l of serum was incubated for 10 minutes in 1000 *μ*l of TE buffer, and 10 *μ*l of NaOH was added to hydrolyze the diacetate and, thus, activate the dichlorofluorescein. Emitted fluorescence was measured with Jasco FP770 equipment. An emission spectrum between 500 and 550 nm was used with each sample. The expressed value is the mean of the emission at 525 nm.

### 2.8. Creatinine Levels

Blood sample was taken as described above. The measurements were processed on a Vitros 5600 Ortho Clinical Diagnostics analytical platform, using the dry chemistry method. Average results are expressed in mg/dl.

### 2.9. Statistical Analysis

The Statistix 7.0 program was used. The Student *t*-test to highlight differences between 2 groups and the ANOVA test for 4 groups were performed. A positive of *p* < 0.05 was used. Pearson test was used to highlight correlation between 2 variables.

## 3. Results

**Figure 2:**
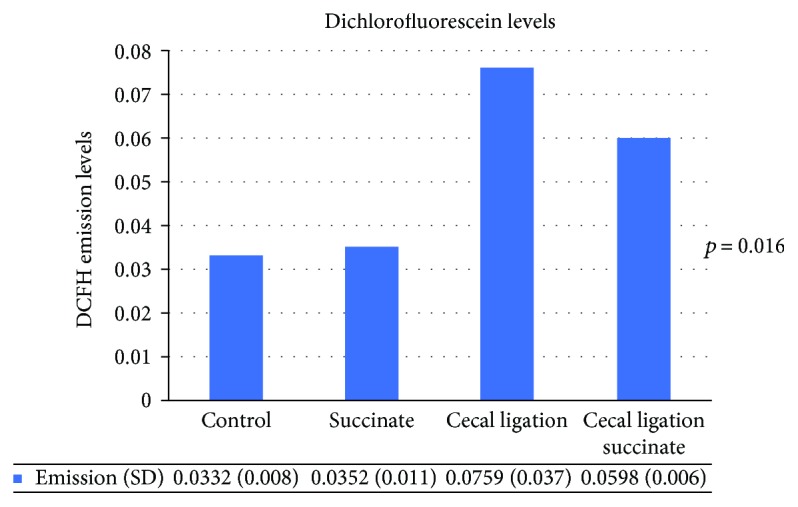
Serum ROS levels. DCFH emission levels. ANOVA test showed differences between groups (*p* = 0.016). There were significant differences between groups 1 and 3 (*p* = 0.007) and groups 3 and 4 (*p* = 0.007). No differences were found between groups 1 and 4 (*p* = 0.3).

DCFH average emissions were as follows: group 1 (0.0332 (SD = 0.008)); group 2 (0.0352 (SD = 0.011)); group 3 (0.0759 (SD = 0.037)); and group 4 (0.0598 (SD = 0.006)) (Figure 2). There were significant differences among the 4 groups (ANOVA test *p* = 0.016). Furthermore, there were significant differences between groups 1 and 3 (*p* = 0.007) and groups 3 and 4 (*p* = 0.007). No differences were found between groups 1 and 4 (*p* = 0.3).

**Figure 3:**
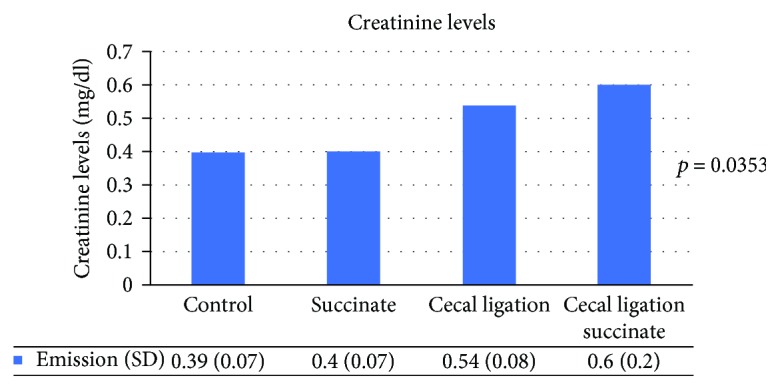
Serum creatinine levels. ANOVA test showed differences between groups (*p* = 0.0353).

Average serum creatinine levels were as follows: group1 (0.39 mg/dl (SD = 0.07)); group 2 (0.4 mg/dl (SD = 0.07)); group 3 (0.54 mg/dl (SD = 0.08)); and group 4 (0.6 mg/dl (SD = 0.2)) (shown in Figure 3). There were also significant differences among the 4 groups (ANOVA test *p* = 0.035), but there were no differences between groups 1 and 3 (*p* = 0.33) or groups 3 and 4 (*p* = 0.07). However, there were differences between groups 1 and 4 (*p* = 0.037). Finally, there were no differences in serum lactate levels among the 4 groups (ANOVA test *p* = 0.3).

**Figure 4:**
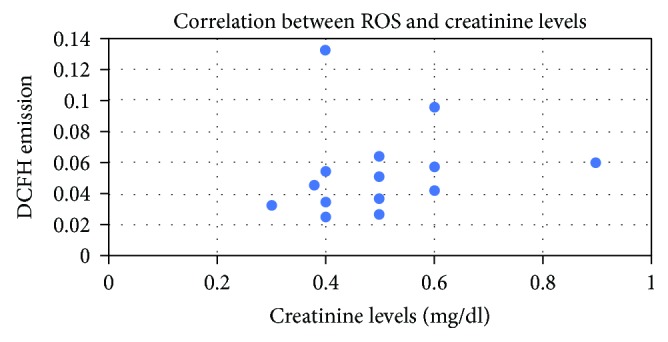
Correlation between DCFH levels and creatinine. There was no correlation between creatinine levels and DCFH emission (rho = 0.24; *p* = 0.3).

On the other hand, there was no correlation in creatinine levels among the 4 groups (rho = 0.24; *p* = 0.3) (Figure 4).

## 4. Discussion

It can be seen in this study that systemic ROS levels were elevated in septic rats, and administering parenteral succinate reduced ROS production in septic animal. Also, succinate did not reduce creatinine levels. At last, creatinine levels did not correlate with DCFH levels.

This study is innovative, as systemic ROS, or serum ROS, were measured in sepsis. Literature describes the presence of ROS at the tissue level and associates tissue damage with the presence of these molecules. In other studies, we measured the presence of ROS in yeast in the presence of Menadione [17]. In the study, we used the DCFH technique to measure the presence of molecules in the serum. The DCFH technique measures ROS, mainly at the intracellular level [18–21], since the fluorophore (Dichlorofluorescein Diacetate) needs diacetate to be separated in order to be activated and emit a signal at 525 nm. The separation of the diacetate takes place inside the cells due to the presence of esterases. This technique is also described for measurements in extracellular fluids. The technique varies in that, during incubation, NaOH is added to separate the diacetate without the need for esterases. This technique has already been described in literature [21–24].

It is known that ROS at the tissue level damage proteins, cell membranes, and nucleic acids. This damage can lead to cell death via apoptosis, which has been described many times. This description is mainly at an experimental level in cell lines or experimental models and at the tissue level. In literature, the presence of systemic ROS in an animal model of sepsis is described, but they are measured with the TBARS technique, and they are also increased in the model, but their presence is not associated with organ failure markers [25]. Furthermore, the total antioxidant capacity was measured in a study on septic patients, in which those who did not survive had a higher total antioxidant capacity and, in turn, this was a marker of 30-day mortality [26]. In this study, systemic ROS were not measured, and their presence was not associated with organ failure markers [26]. In a different study, ROS in whole blood were measured in septic patients with DCFH. It was shown that patients with a SOFA score greater than 7 had higher ROS levels [27].

*In vivo*, ROS are produced through different pathways, in different cells and in different amounts. Phagocytic cells are the main producers of ROS in acute illness, as a component of immune defenses which its objective is destroying microorganisms [2]. Under physiologic conditions, there is a continuous mitochondrial production of ROS and more precisely in the electron transport chain, which produces 90% of these molecules [3]. There is a clear relation between inflammatory molecules and oxidative stress levels though [2, 7].

This study is also innovative, as systemic ROS levels were reduced in septic rats with succinate, a Krebs cycle intermediate, with no changes in nonseptic rats. This could be due to the fact that the electron transport chain is not affected in nonseptic rats; the effects of the drug are not the same as those seen in septic rats.

Chouchani et al. [28], in their work, express that there is succinate accumulation during hypoxia-reoxygenation. Through several tests, they show that glucose, palmitate, glutamine, and GABA do not contribute to the accumulation. Instead, the origin of the accumulation is fumarate. The reactions that carries out from the formation of citric acid to succinate are all with negative *standard free-energy difference* (*∆G*°′), which means that they are spontaneous. Instead, the reactions go after succinate are with *∆G*°′ = 0 (from succinate to fumarate) or positive *∆G*°′, which means that it is not spontaneous. The reaction from succinate to fumarate will go in diverse ways depending on the concentration of the substrates [29]. During hypoxia, electron transport chain is stopped because there is no final substrate, oxygen. There is no reoxidation of NAD+ and FAD, and anaerobic glycolysis occurs, producing lactate. Also, as there is no NAD+ and FAD, *β*-oxidation is also stopped, so there will be no acetyl-CoA for Krebs. But hypoxia-reoxygenation is not the only physiopathological issue in sepsis.

In sepsis, other mechanisms take place in the mitochondria. Several studies showed that there is low activity in the electron transport chain, in different complex. Lorente et al. showed lower activity of complex IV in platelets of septic patients [30], while Brealey et al. [31] showed a lower activity in complex I, while in complexes II, III, and IV, there was no difference. The same group, also, showed that complex II/III activity remained unchanged in both the muscle and the liver of septic rats [32]. Also, both skeletal muscle and liver complex I activity fell with increasing disease severity in septic rats [32].

In sepsis, electron transport chain activity and mitochondrial respiration are decreased, shown by the lower levels in oxygen consumption and the decrease in ATP levels and ATP/ADP ratio [33]. Also, several metabolic pathways have been proposed as targets in sepsis, such as the pathways that regulate glycolysis or fatty acid metabolism, sources of Acetyl-coA, substrate of Krebs cycle [33]. Succinate has shown to improve mean survival time in septic rats with succinic acid infusion [9, 10]. Also, mitochondrial respiration was augmented in moderately-to-severely septic animals, shown by the recovery of oxygen consumption [8]. In the hepatocytes of lipopolysaccharide-injected rats, the infusion of succinate improved the plasma concentration of free fatty acids and B-hydroxybutyrate, the liver ATP content, and the oxidation of D-glucose as well as the pyruvate/lactate ratio [10].

Several attempts have been made to reduce oxidative stress in septic patients, most of them with inconclusive results [34–37], being parenteral succinate an alternative therapeutic option.

All these results might show that succinate in sepsis can improve systemic ROS levels restoring the electron transport chain, but this does not improve renal function measured with creatinine levels. Also, there is no correlation between systemic ROS and creatinine levels, indicating that ROS production and kidney damage are not linked. At last, we showed [38] that septic patients in the ICU do not have more ROS in the bloodstream than healthy controls, and those patients who died in the ICU do not have more systemic ROS than those who survived.

In this study, no correlation between systemic ROS levels and creatinine was found. Sepsis is the most common cause of kidney failure in the ICU [39]. This can be a pathophysiologic mechanism of distant injury in sepsis or a severity marker of the disease. Septic rats showed higher creatinine levels, which were not reduced with the administration of succinate. This can be due to the fact that the uncoupling of the electron transport chain and the production of ROS are not part of the pathophysiology or due to the fact that creatinine is a late marker of kidney injury, and the duration of the test was not enough to show improvement. There are other earlier and more accurate biomarkers of kidney injury, such as NGAL, which is not routinely used yet [40–42]. Therefore, we chose creatinine as a marker of kidney injury.

The question that needs to be solved is that do ROS cause tissue damage or just molecules that show that mitochondrial processes are not working correctly. More studies are needed to solve this question.

## 5. Conclusions

Parenteral succinate reduces systemic ROS levels, but it does not reduce serum creatinine levels. Further studies are needed to understand this drug's mechanism of action.

## Data Availability

The data used to support the findings of this study are available from the corresponding author upon request.
